# Queuine tRNA ribosyltransferase 1 deficiency ameliorates hepatic steatosis and atherosclerosis possibly via modulating lipogenesis

**DOI:** 10.1016/j.gendis.2026.102073

**Published:** 2026-02-07

**Authors:** Runda Wu, Shuning Zhang, Wanxin Wang, Zheng Dong, Wei Gao, Yongduan Teng, Yuxiang Dai, Shangyu Hong, Kang Yao, Junbo Ge

**Affiliations:** a Department of Cardiology, Zhongshan Hospital, Fudan University, Shanghai 200032, China; b Shanghai Institute of Cardiovascular Disease, Shanghai 200032, China; c State Key Laboratory of Genetic Engineering, School of Life Sciences and Human Phenome Institute, Fudan University, Shanghai 200438, China; d Department of Cardiology, Nanjing Drum Tower Hospital, Nanjing, Jiangsu 210008, China; e Key Laboratory of Viral Heart Diseases, National Health Commission, Shanghai 200032, China; f Key Laboratory of Viral Heart Diseases, Chinese Academy of Medical Sciences, Shanghai 200032, China; g National Clinical Research Center for Interventional Medicine, Shanghai 200032, China; h Institutes of Biomedical Sciences, Fudan University, Shanghai 200032, China

Atherosclerotic cardiovascular disease (ASCVD) is the leading cause of morbidity and mortality in patients with metabolic dysfunction-associated fatty liver disease (MAFLD).^1^ Liver steatosis along with hyperlipidemia originates primarily from disturbed lipid metabolism in the liver and confer major risks for atherosclerosis and adverse cardiovascular events. Elevated hepatic *de novo* lipogenesis (DNL) contributes to steatosis and hyperlipidemia, and strategies to suppress DNL have displayed the potential to attenuate atherosclerotic lesions.^2^ Queuine tRNA ribosyltransferase 1 (QTRT1) is an enzyme responsible for tRNA modification by substitution of guanosine with queuosine at the anticodon wobble position. Our previous study found that QTRT1 mutations influenced ASCVD risk through DNL dysregulation and accelerated atherosclerosis.^3^ However, the role and underlying mechanism of QTRT1 inhibition in liver steatosis induced by various factors and in atherosclerosis remain unclear.

Here, we investigated the role of QTRT1 deficiency in lipid metabolism using several experimental mouse models of liver steatosis and atherosclerosis. First, based on an established liver-specific QTRT1 knockout mouse (LKO) model (Fig. S1L, S1M), we overexpressed PCSK9 by adeno-associated viruses (AAVs) in these LKO mice and their littermate control (flox/flox). After 12 weeks on a high-fat and high-cholesterol (HFHC) diet, both serum and hepatic levels of triglyceride (TG), total cholesterol (TC) and low-density lipoprotein-cholesterol (LDL-c) were markedly decreased in LKO mice at sacrifice (Fig. 1A and B). These findings are in accordance with Oil Red O (ORO) and hematoxylin and eosin (H&E) staining of liver sections, which showed that lipid accumulation was mitigated in LKO mice compared with control mice (Fig. 1C). QTRT1 deficiency resulted in fewer arterial plaques and less plaque burden, and seemed to contribute to smaller necrotic core size and F4/80-positive area, and larger Picrosirius Red-positive area (Fig. 1D), which indicates a more stable atherosclerotic lesion with more collagen covered as a solid fibrous cap and fewer infiltration with inflammatory macrophages as necrotic foam cells. The instability of atherosclerotic plaques usually features large lipid cores covered by a thin fibrous cap, which denotes risks of plaque rupture and adverse events.^4^ The ameliorated plaques in LKO mice was in line with the lower serum levels of LDL-c and TG (Fig. 1E), emphasizing the role of QTRT1 deficiency in improvement of both liver steatosis and atherosclerosis.

**Figure 1:**
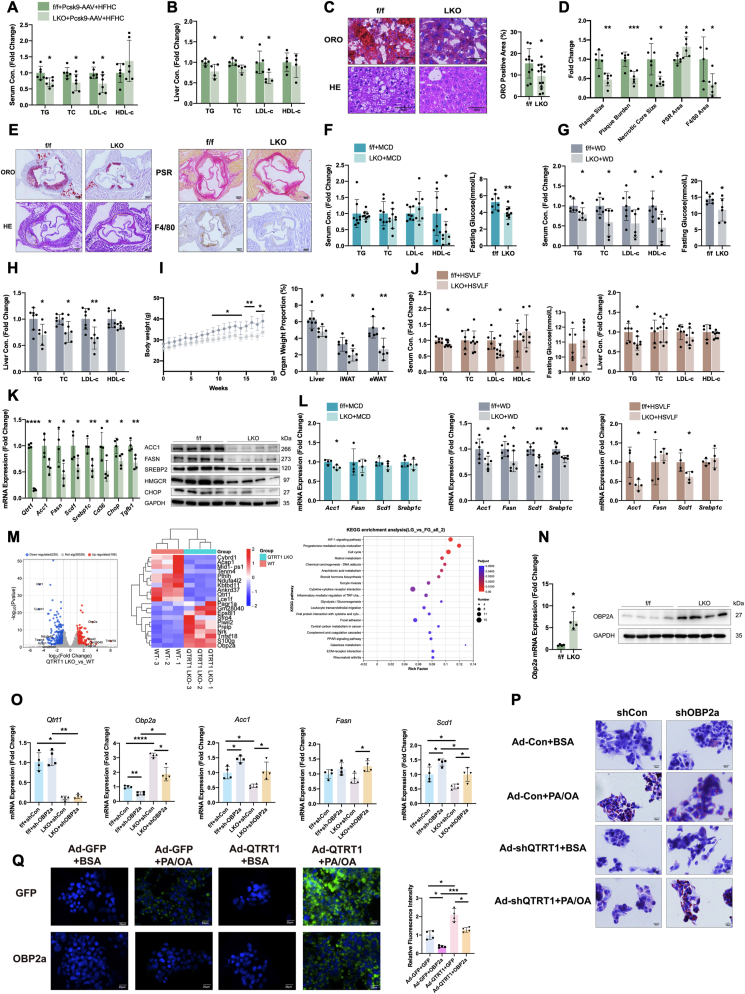
Queuine tRNA Ribosyltransferase 1 (QTRT1) deficiency ameliorates hepatic steatosis and atherosclerosis by modulating lipogenesis. **(A, B)** Serum and hepatic levels of triglycerides (TG), total cholesterol (TC), low-density lipoprotein-cholesterol (LDL-c) and high-density lipoprotein-cholesterol (HDL-c) in liver-specific QTRT1 knockout mice (LKO) and control flox/flox littermates (f/f) which were both injected with gain-of-function AAVs of mouse PCSK9 and fed a high-fat and high-cholesterol (HFHC) diet for 12 weeks; *n* = 4^_^6 for each group of mice. Con. denotes concentration. **(C)** Representative images of Oil Red O (ORO) and H&E-stained liver sections of LKO and f/f mice injected with PCSK9-AAVs and fed HFHC, obtained at 20 × magnification along with corresponding histological measurements of steatosis by percentage of ORO staining-positive area; *n* = 5^_^7 for each group of mice (2 different sections chosen for each mouse). Scale bar = 50 μm. **(D, E)** Quantification of total plaque size depicted as ORO-positive area in the aortic root sections of LKO and f/f mice injected with PCSK9-AAVs and fed HFHC, plaque burden as a percentage of lumen area of aortic section, necrotic core size depicted by yellow dotted lines as a percentage of plaque size, picrosirius red (PSR)-stained area and F4/80-positive area as a percentage of plaque size, and representative images of aortic roots stained with ORO, HE, PSR and F4/80 taken at 5 × magnification; *n* = 6 for each group of mice. Scale bar = 200 μm. **(F)** Levels of serum TG, TC, LDL-c and HDL-c as well as fasting glucose levels of LKO and f/f mice fed on a methionine- and choline-deficient (MCD) diet for 8 weeks at sacrifice; *n* = 8^_^9 for each group of mice. **(G, H)** Levels of serum and hepatic TG, TC, LDL-c and HDL-c as well as fasting glucose levels of LKO and f/f mice fed on a Western diet (WD) for 18 weeks at sacrifice; *n* = 6^_^7 for each group of mice. **(I)** Bodyweight curve of LKO and f/f mice fed on WD and weight of organs including liver, inguinal white adipose tissue (iWAT) and epididymal WAT(eWAT) indicated as a proportion of bodyweight at sacrifice; *n* = 6^_^7 for each group of mice. **(J)** Levels of serum and hepatic TG, TC, LDL-c and HDL-c as well as fasting glucose levels of LKO and f/f mice fed on a high-sucrose and very low-fat (HSVLF) diet for 12 weeks at sacrifice; *n* = 6^_^8 for each group of mice. **(K)** Levels of mRNA and protein expression of main genes regulating lipid synthesis and endoplasmic reticulum stress in LKO mice injected with PCSK9 AAVs and fed with HFHC diet; *n* = 4 for each group of mice. **(L)** Levels of mRNA expression of main genes of lipogenesis in other mice models, *Acc1, Fasn, Scd1* and *Srebp1c*; *n* = 4^_^7 for each group of mice. **(M)** Volcano plot with fold change, heatmap of 10 top up- and downregulated genes and KEGG pathway-enrichment analysis by RNA sequencing of primary hepatocytes isolated from LKO and f/f mice; *n* = 3 for biological repeats. **(N)** mRNA and protein expression levels of *Obp2a* in the livers of LKO and control mice injected with PCSK9-AAVs on an HFHC diet; *n* = 4 for each group of mice. **(O)** mRNA expression levels of *Obp2a, Qtrt1,* and genes regulating lipogenesis in primary hepatocytes isolated from LKO and f/f mice, infected with adenoviruses packaging shRNA of OBP2A (shOBP2a) or nonsense sequence (shCon) for 48 h; *n* = 4 for biological repeats. **(P)** Representative images of ORO staining of HepG2 cells transfected with plasmids of shOBP2a and shCon for 48 h, followed by infection with adenoviruses packaging shRNA of QTRT1 (Ad-shQTRT1) and control sequence (Ad-Con) and treated with palmitic acid and oleic acid (PA/OA, 100 and 200 μM) or bovine serum albumin (BSA) as control solvent for 24 h taken at 50 × magnification; *n* = 3 for biological repeats. Scale bar = 20 μm. **(Q)** Merged immunofluorescence images and quantification of BODIPY staining in HepG2 cells transfected with plasmids expressing coding sequence of OBP2A or GFP for 48 h and then infected with adenoviruses overexpressing QTRT1 (Ad-QTRT1) and GFP control (Ad-GFP) and treated with PA/OA for 24 h, BODIPY for labeling lipid droplets and DAPI for labeling cell nuclei; *n* = 4 for biological repeats. Scale bar = 20 μm. Data are presented as the mean ± SD. Statistical significance (∗*p* < 0.05, ∗∗*p* < 0.01, ∗∗∗*p* < 0.001, ∗∗∗∗*p* < 0.0001) was determined using unpaired *t*-test for data conforming to a normal distribution (A–N), or Mann–Whitney *U* test for not passing a normal distribution test (part of data from G, J and L), or one-way ANOVA test for comparison among three or more groups (O and Q). During RNA sequencing analysis, genes that were significantly changed by > 2-fold with an FDR-adjusted *p* value < 0.05 were selected as differential expression genes.

Next, we tested whether QTRT1 deficiency would play a role in commonly used models of MAFLD and metabolic dysfunction-associated steatohepatitis (MASH). When fed with a methionine- and choline-deficient (MCD) diet for 8 weeks to establish a MASH animal model, compared to control mice, only serum high-density lipoprotein-cholesterol (HDL-c) and fasting glucose levels were alleviated in LKO mice (Fig. 1F). In contrast, serum and hepatic levels of TG, TC and LDL-c, and serum levels of HDL-c were decreased in LKO mice fed on a Western Diet (WD) for 18 weeks which is used to conduct a MAFLD model (Fig. 1G and H), similar to the reduced lipid levels in HFHC diet (Fig. 1A and B). Similar decline of fasting glucose caused by QTRT1 inhibition was also displayed in mice fed on WD (Fig. 1G) and HFHC (Fig. S1J). Marked decrease of bodyweight and organ weight was found in LKO mice fed on WD (Fig. 1I), while no significant change was uncovered in mice fed on HFHC and MCD diet (Fig. S1E–S1H, S2A–SC). Both MASH and MAFLD models did not show a difference in the serum levels of alanine transaminase and aspartate transaminase (Figs. S2D, S2E, S3A, S3B). These data suggested a major benefit of QTRT1 ameliorating liver steatosis, overweight and hyperlipidemia caused by high-fat diet, but a partly different effect in steatohepatitis model.

Given the decreased levels of fasting glucose in LKO mice, we further test whether loss of QTRT1 would also benefit from a high-carbohydrate diet. For this purpose, mice were fed with a high-sucrose and very low-fat (HSVLF) diet for 12 weeks, which often causes adiposity and hepatic steatosis. It was found that QTRT1 deficiency resulted in a similar but milder reduction of hepatic TG, serum TG and LDL-c (Fig. 1J). In contrast, the fasting glucose and transaminase levels or bodyweight were not influenced (Fig. 1J; Fig. S4A–S4E). qPCR results suggested primary downregulation of genes related to DNL such as *Acc1*, *Scd1*, *Fasn* and *Srebp1c* in LKO mice fed a HFHC and injected with the PCSK9 AAVs (Fig. 1K), which is in line with our previous findings.^3^ No changes were observed in *Qtrt2* expression (Fig. S1K), and this downregulation was almost universal among other models for liver steatosis (Fig. 1L). QTRT1 deficiency also induced downregulated expression of *Cd36*, *Tgf-β1 and Chop* which is critical in endoplasmic reticulum stress (Fig. 1K). Notably, genes related to lipoprotein transportation, fatty acid oxidation, inflammatory factors and other upstream transcription factors did not show significant differences (Fig. S5A–S5E). At the protein level, enzymes responsible for DNL (ACC1 and FASN) were downregulated, while factors inducing cholesterol synthesis (SREBP2 and HMGCR) and CHOP showed a downward trend in LKO mice (Fig. 1K; Fig. S7A). In addition, QTRT1 mRNA expression remained unchanged in human samples of liver steatosis or atherosclerosis (Fig. S6A and S6B). These observations suggested that QTRT1 was essential in lipid metabolism and atherosclerosis, while its role in glucose metabolism was still uncertain.

To explore the underlying mechanism, we conducted RNA sequencing of primary hepatocytes isolated from LKO and control mice. Differential expression gene analysis demonstrated that among 454 genes 199 were upregulated and 255 were downregulated. KEGG pathway analysis indicated that retinol metabolism and sterol hormone biosynthesis were significantly changed after inhibition of QTRT1 (Fig. 1M). Among these genes, *OBP2A* coding Odorant-binding protein 2A (OBP2A) was significantly upregulated in the livers of LKO mice (Fig. 1M and N), and it was previously reported to be associated with glucose metabolism and regulating lipid synthesis during liver steatosis.^5^ Given that OBP2A decreased liver lipid levels in mice by inhibiting hepatic lipogenesis,^5^ which was in line with the results of LKO mice, we hypothesized that the amelioration of liver steatosis and lipogenesis by loss of QTRT1 was mediated by upregulation of OBP2A. We knocked down OBP2A by infection of hepatocytes derived from LKO and control mice with adenoviruses expressing shRNA targeting OBP2A (shOBP2A, Fig. 1O). Compared with hepatocytes from f/f mice, those from LKO mice showed decreased expression of *Acc1* and *Scd1*, which was inhibited after knockdown of OBP2A. It turned out that inhibition of OBP2A could reverse the mitigated size and amount of lipid droplets stained by ORO in cells with downregulated QTRT1 (Fig. 1P). Conversely, the aggravated accumulation of lipids stained by BODIPY via QTRT1 was relieved by further upregulation of OBP2A (Fig. 1Q). These results confirmed that OBP2A was critical to downregulate lipogenesis induced by deficiency of QTRT1.

Combined with *in vivo* and *in vitro* studies, we provided evidence that QTRT1 inhibition effectively attenuated liver steatosis, hyperlipidemia, and atherosclerotic lesions. Notably, the inhibition of QTRT1 could confer potential adverse side-effects, as tRNA modification catalyzed by QTRT1 is essential in general protein translation and fidelity, which warrants further investigation. OBP2A influenced lipogenesis and might mediate the effect of QTRT1 deficiency on hepatic lipid accumulation. These findings might support the further clinical design and investigation of specific QTRT1 inhibitors to treat both MAFLD and ASCVD.

## CRediT authorship contribution statement

**Runda Wu:** Writing – original draft, Project administration, Investigation, Formal analysis, Data curation, Conceptualization. **Shuning Zhang:** Methodology, Funding acquisition, Data curation. **Wanxin Wang:** Investigation. **Zheng Dong:** Investigation, Data curation. **Wei Gao:** Formal analysis, Data curation. **Yongduan Teng:** Investigation. **Yuxiang Dai:** Writing – review & editing, Supervision, Methodology, Conceptualization. **Shangyu Hong:** Writing – review & editing, Supervision, Methodology, Conceptualization. **Kang Yao:** Writing – review & editing, Supervision, Data curation, Conceptualization. **Junbo Ge:** Supervision.

## Ethics declaration

All animal experiments were performed according to ethical standards and the study protocol was approved by the Central Ethics Committee of Zhongshan Hospital, Fudan University (approved number: 2019-180).

## Funding

This work was supported by the Clinical Research Plan of the Shanghai Hospital Development Center, China (No. SHDC2020CR1007A), Shanghai Municipal Science and Technology Major Project, China (No. 2017SHZDZX01), the 10.13039/501100001809National Natural Science Foundation of China (No. 82170460), Shanghai Municipal Key Clinical Specialty, China (No. shslczdzk01701), Shanghai Top Priority Research Center Construction Project, China (No. 2022ZZ01010) and the Natural Science Foundation of Shanghai, China (No. 21ZR1412700, 21ZR1413500). Dr. Hong was supported by the 10.13039/501100012166National Key Research and Development Program of China (No. 2019YFA0802300), Training Program of the Major Research Plan of the 10.13039/501100001809National Natural Science Foundation of China (No. 91957117), and the 10.13039/501100001809National Natural Science Foundation of China (No. 31971082). Dr. Dai was supported by the National Key Research and Development Project of China (No. 2021YFC2500500, 2023YFC2506500) and the 10.13039/501100012247Program of Shanghai Academic Research Leader, China (No. 22XD1423300). Dr. Wu received funding from the 10.13039/501100002858China Postdoctoral Science Foundation (No. 2023M730667) and the Outstanding Resident Clinical Postdoctoral Program of Zhongshan Hospital Affiliated to Fudan University, China (No. 2023ZSQN32, 2024ZYYS-004).

## Conflict of interests

None.
