# Supplementary material for: Queuine tRNA ribosyltransferase 1 deficiency ameliorates hepatic steatosis and atherosclerosis possibly via modulating lipogenesis
Source: Genes Dis. 2026 Feb 7;13(6):102073. doi: 10.1016/j.gendis.2026.102073 (PMC13380712; doi:10.1016/j.gendis.2026.102073)
Supplement: Multimedia component 1 [file mmc1.docx]

**Supplementary material file**

**Queuine tRNA Ribosyltransferase 1 Deficiency Ameliorates Hepatic Steatosis and Atherosclerosis Possibly via Modulating Lipogenesis**

Runda Wu, Shuning Zhang, Wanxin Wang, Zheng Dong, Wei Gao, Yongduan Teng, Yuxiang Dai^*^,Shangyu Hong^*^, Kang Yao^*^ and Junbo Ge

*Correspondence authors (Emails):

shangyu_hong@fudan.edu.cn,

[dai.yuxiang@zs-hospital.sh.cn](mailto:dai.yuxiang@zs-hospital.sh.cn)

[yao.kang@zs-hospital.sh.cn](mailto:yao.kang@zs-hospital.sh.cn),

This file contains:

**Supplementary Methods**

**Supplementary Figures 1-7**

**Supplementary Table 1**

**References**

**Supplementary Methods**

- 1. Animal experiments

All animal experiments were performed according to ethical standards and the study protocol was approved by the Central Ethics Committee of Zhongshan Hospital, Fudan University (approved number: 2019-180). All procedures were carried out in adherence with the guidelines for ethical review of laboratory animal welfare in China and guidelines of NIH. Eight-week-old male WT (*Qtrt1^fl/fl^*, GemPharmatech Co., T015234) and heterozygous Alb-Cre (Alb-Cre^+/–^, GemPharmatech Co., T064038) mice were bred to generate Qtrt1LKO (*Qtrt1^fl/fl^* Alb-Cre^+/–^) mice. These mice were fed a high-fat and high-cholesterol (HFHC) diet (D12108c, 40% kcal fat, 40% kcal carbohydrate, and 20% kcal protein, 1.25% cholesterol added; Research Diet Inc.) for 12 weeks. Specifically, WT and Qtrt1LKO mice (8-week-old) were injected with a single dose of Pcsk9-D377Y-AAV8 (10^11^ vg per mouse, WZ Biosciences Inc.) to enhance PCSK9 expression via tail injection. All mice were housed in a specific pathogen-free and climate-controlled environment with a 12-h light-dark cycle and provided free access to food and water. Eight-week-old WT and Qtrt1LKO male mice were also fed a high-sucrose, very low fat diet (HSVLF) (PD23041401, 2.6% kcal fat, 76.7% kcal carbohydrate, and 20.69% kcal protein; SYSE Biotech) for 12 weeks, methionine- and choline-deficient diet (MCD)(A02082002BR, SYSE Biotech) for 8 weeks, and a high-fat Western Diet (WD) (TD120528, SYSE Biotech) for 18 weeks. The reason we chose to construct these mice models is to discover different phenotypes of QTRT1 deficiency under various disease patterns with distinctive dietary patterns. We also utilized previously bred and hepatic-specific genetically deficient mice as we would like to focus on the lipid metabolism which mainly takes place in the liver. The reason we adopted the PCSK9 function-enhanced AAV and dietary intervention by HFHC to establish atherosclerosis was referred to several previous studies,^1-3^ and we used MCD, WD, and HSVLF diet to construct mice models of NASH,^4^ NAFLD^5^ according to various widely-recognized studies.^6^ Body mass and food intake were measured weekly, and fasting glucose and serum lipids were measured every 4 weeks after fasting for 4 h by blood collection through tail veins. After fed with different diets for certain duration in various models of metabolic dysfunction, mice were fasted and anesthetized using an intraperitoneal injection of pentobarbital sodium (60 mg kg^-1^), and euthanized by cervical dislocation for blood and organ tissue samples.

- 1. Blood biochemical analysis

Triglyceride, cholesterol, LDL-C, and HDL-C levels in mouse serum were measured using kits from the Nanjing Jiancheng Bioengineering Institute (A110-1-1, A111-1-1, A113-1-1, and A112-1-1, respectively), following the manufacturer’s instructions. Serum alanine transaminase (ALT) and aspartate transaminase (AST) levels were measured using kits from the Nanjing Jiancheng Bioengineering Institute (C009-1-1 and C010-1-1).

- 1. Liver lipid extraction and analysis

Livers were transferred from -80 °C to liquid nitrogen and chopped to 20–40 mg size with a blade. The livers were transferred into Eppendorf tubes containing 1 mL of pre-cooled 2:1 chloroform: methanol, followed by tissue homogenization at 2,100 rpm for 4 min. The homogenate was transferred into glass screw-top tubes that had been dried off the solvent, and another 2 mL of 2:1 chloroform:methanol was added before rinsing the residual homogenate. After overnight incubation at room temperature, liver lipids were extracted when tissues sank to the bottom of the tube, and 0.9% NaCl solution (0.2 fold of total volume) was added and centrifuged at 2000 ×*g* at 25 °Cfor 10 min. The lipid extract sample was transferred to a new tube and dried under a nitrogen atmosphere. Then, 1.5 mL/150 mg of 0.6:0.267:0.133 n-butanol: Triton X-100: methanol was added and vortexed, followed by similar measurements of serum lipids using the same kits and standardized by sample weight.

- 1. Immunohistochemical, H&E, Picrosirius Red, and Oil-Red-O staining

Immunohistochemical staining of the vessel plaques was conducted as previous studies ^7^. Briefly, the liver and heart tissues were fixed in 4% paraformaldehyde for 24 h and then frozen embedded with the thickness of the frozen section being 8–10 μm. Aortic arch sections were incubated with F4/80 (1:50 dilution, Thermo Fisher, 14-4801-82). After washing with PBS, the sections were incubated with secondary antibodies. Liver and aortic arch sections were also stained with H&E (Servicebio), picrosirius red (Servicebio), and Oil Red O (Sigma), as previously reported ^7;8^. Images were analyzed using the ImageJ 1.54f software.

- 1. Cell experiments
     1. Cell isolation and culture

Primary hepatocytes were isolated and cultured as previously described.^9^ Briefly, primary hepatocytes were isolated from 8-week-old mice anaesthetized using 1% pentobarbital sodium (50mg/kg) by a single dose of intraperitoneal injection, and perfused via the post cava with buffer and a subsequent digestion solution containing collagenase type I (C0130; Sigma). The digested liver was excised, minced, filtered, and centrifuged, and the cells were seeded in plates pre-coated with collagen type I solution from the rat tail (Sigma, C3867). During the procedure, mice were euthanized with cervical dislocation. Human HepG2 hepatocytes (TCHu 72; National Collection of Authenticated Cell Cultures of China) were cultured in DMEM (Gibco) with 10% FBS, 50U mL^-1^ penicillin and 50 μg mL^-1^ streptomycin at 37 °C and 5% CO_2_. Then, infection with adenoviruses for 48 h, the total RNA of the hepatocytes was harvested for qPCR. In HepG2 cells, after infection with adenoviruses or transfection with plasmids, palmitic acid (Sigma, treatment concentration at 100 μM) and oleic acid (Sigma, at 200 μM) dissolved in bovine serum albumin (Sigma) were added, and 24 h after treatment, cells were rinsed by PBS and fixed by 4% paraformaldehyde for additional staining by Oil Red O and immunofluorescent BODIPY as previously described ^10^. Images were analyzed using the ImageJ 1.54f software.

- - 1. Cell transfection and infection

Adenoviruses for overexpression of QTRT1 and GFP were purchased from WZ Biosciences Inc. Adenoviruses for QTRT1 were purchased from WZ Biosciences Inc.. shRNA and overexpression plasmids targeting OBP2A and adenovirus shRNA were purchased from Shanghai GeneChem. The shRNA sequences used were: sh-OBP2A: CCGGGCATGAATCTCCGACTGGAAACTCGAGTTTCCAGTCGGAGATTCATGCTTTTTG; the sequence of sh-QTRT1 was the same as the sequence in our previous study ^9^.

Primary hepatocytes from the mice were infected with adenoviruses for 48 h at an MOI of 10. Human HepG2 cells were also infected with adenoviruses or transfected with plasmids using the Lipofectamine 3000 kit (Thermo Fisher Scientific) for 48 h. For experiments, HepG2 cells were first transfected with plasmids for 48 h when the medium was changed daily and then infected with adenoviruses for another 48 h. After treatment, total RNA or proteins in the hepatocytes were harvested for further analyses.

- 1. mRNA and real-time PCR

RNA was extracted using TRIzol Reagent (Thermo Fisher Scientific), and real-time PCR assays were performed using Taq Pro Universal SYBR qPCR Master Mix (Vazyme, Q712-02) on a QuantStudio 7 flex sequence detection system following the manufacturer’s instructions. For RNA extraction from human whole blood, the QIAamp RNA Blood Mini Kit (QIAGEN) was used according to the manufacturer’s instructions. The primers used are listed in the Supplementary Data section. mRNA levels in different groups were recorded using the 2^-ΔΔCt^ method. For most analyzes, TATA-box binding protein (TBP) was the internal control, and for testing expression of *SCD1* in other tissues, glyceraldehyde-3-phosphate dehydrogenase (GAPDH) served as the internal control. PCR thermal cycling conditions were 95 ℃for 30 s, followed by 40 cycles of 95 ℃ for 10 s and 60 ℃ for 30 s.

- 1. RNA sequencing and analysis

To perform RNA-seq, total RNA was isolated from primary hepatocytes of LKO (n = 3) and control mice (n = 3). cDNA libraries were constructed using the Illumina TruSeq RNA Library Prep Kit (RS-122-2001), following the manufacturer’s instructions. RNA-seq was performed using an Illumina NovaSeq 6000 sequencer (San Diego, CA, USA). Sequencing data were processed using the BaseSpace Sequence Hub software. Sequenced reads were trimmed to remove adaptor sequences and mapped to the mouse reference genome using HISAT2 software. The mapped reads of each sample were assembled using StringTie using a reference-based approach. Genes that were significantly changed >2-fold with an FDR-adjusted P-value <0.05 were selected for further analyses. In addition, functional enrichment analyses, including GO and KEGG, were performed to identify which DEGs were significantly enriched in GO terms and metabolic pathways at a Bonferroni-corrected P-value < 0.05, compared to the whole-transcriptome background. GO functional enrichment and KEGG pathway analyses were performed using GoATools and Python Science, respectively.

- 1. Western blotting

Frozen livers were minced and grinded in a mixed buffer containing RIPA Lysis buffer (Beyotime, P0013B) and proteinase inhibitors (Beyotime, P1005). Lysates were incubated at 4 °C for 60 min, and supernatants were collected after centrifugation at 10,000 ×*g* for 30 min. Supernatants were used for western blotting and incubated with a primary antibody against QTRT1 (Santa Cruz, sc398918), ACC1 (Abclonal, A19627), FASN (Abclonal, A0461), SREBP2 (Abclonal, A13049), HMGCR (Abclonal, A19063), CHOP (Abclonal, A21902), OBP2A (R&D, AF7974), and GAPDH (Sigma, G8795-200) at 4 ℃ overnight. Afterwards, it was incubated with secondary antibody and presented for blotting. The anti-GAPDH antibody was used at a 1:5,000 dilution, and other primary antibodies were used at a 1:1000 dilution.

1.9 DEG analysis in Gene Expression Omnibus datasets

Transcriptome sequencing datasets in the study were collected from the Gene Expression Omnibus (GEO) (<http://www.ncbi.nlm.nih.gov/gds/>) database and analyzed using the online software GEO2R (<http://www.ncbi.nlm.nih.gov/geo/geo2r/>), and the GEO datasets used in the study included GSE63067 and GSE28829.

- 1. Statistical information

GraphPad Prism 9.0.0 (Graphpad) was used for statistical analysis. All data are expressed as mean ± SD. The Shapiro-Wilk normality test was used to assess the distribution. Comparisons between data of two separate groups were performed using t-test for data conforming to a normal distribution or Mann–Whitney U test for not passing a normal distribution test. When the variance was equal between two groups, Student’s t-test was utilized, otherwise Welch’s t-test was used. Comparison among three or more groups were conducted using one-way ANOVA followed by Tukey’s or Bonferroni’s multiple comparison test to evaluate differences among multiple groups. When the F-test indicated unequal standard deviations, Brown-Forsythe and Welch ANOVA tests were used to correct the analysis. Statistical significance was set at *p <* 0.05. For the RNA-seq study, an FDR-adjusted P value was calculated after correction for multiple testing, and genes that were significantly changed >2-fold with an FDR-adjusted P-value <0.05 were selected for further analyses, as has mentioned above.

**Supplementary Figures**


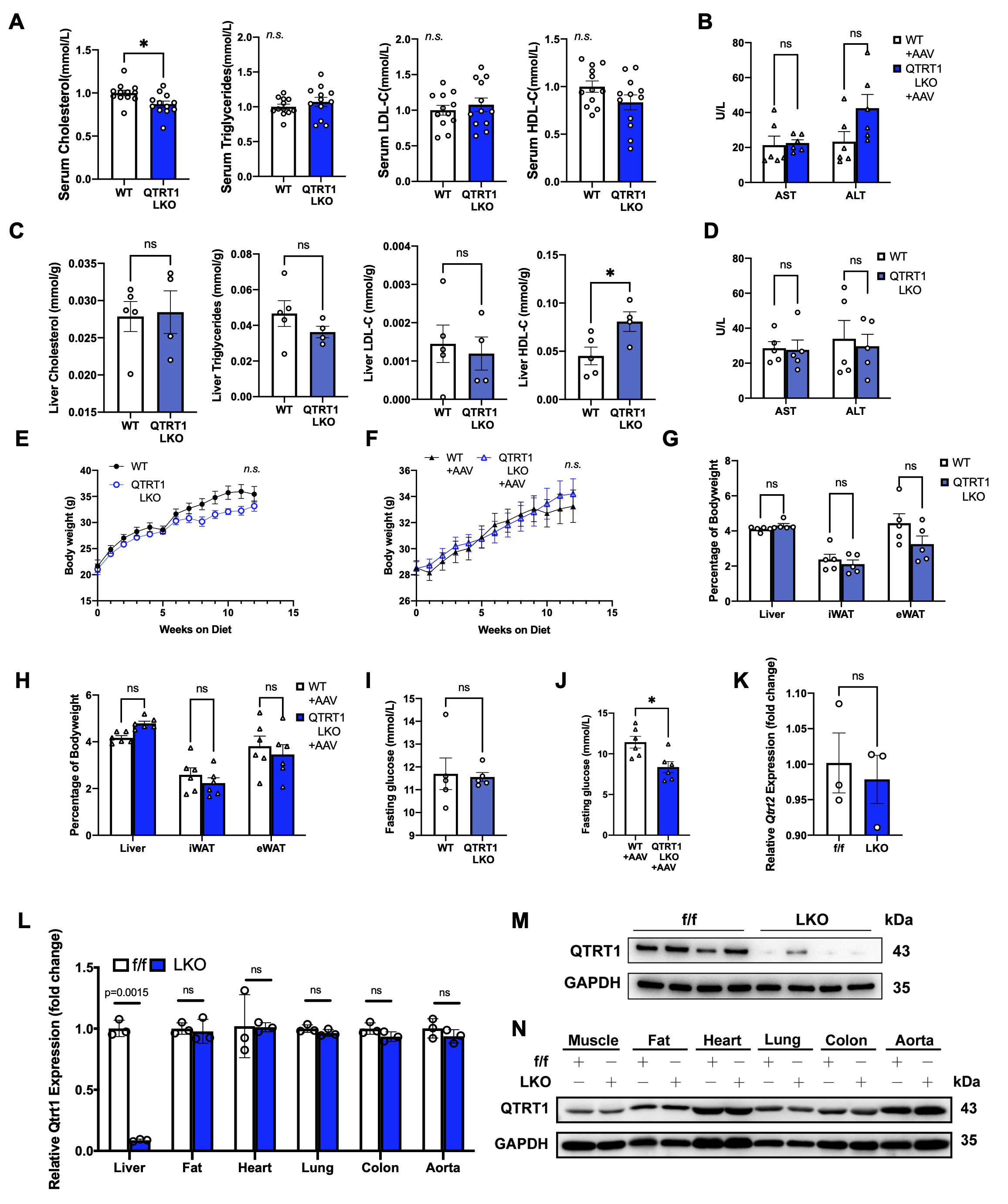


**Supplementary Fig. 1. Liver-specific deletion of QTRT1 protected mice against steatosis and hyperlipidemia when fed a high-fat and high-cholesterol diet (HFHC).**

(A) Serum cholesterol, triglyceride, LDL-C, and HDL-C levels in LKO mice and f/f control mice (denoted as “WT”) before feeding with the HFHC diet (n = 12 for each group of mice).

(B) Serum ALT and AST levels in LKO mice injected with Pcsk9-D377Y-AAV8 and control mice at euthanization (n = 6 for each group of mice).

(C) Liver lipids of LKO mice not injected with AAV at euthanization (n = 4^_^5 for each group of mice).

(D) Serum ALT and AST levels in LKO mice that were not injected with AAV and their counterpart control mice (n = 5 for each group of mice).

(E-F) Body weight of mouse cohorts at euthanization (n = 5^_^6 for each group of mice).

(G-H) Organ mass ratio calculated by the proportion of organ mass in mouse cohorts at euthanization (n = 5^_^6 for each group of mice).

(I-J) Fasting glucose levels at euthanization (n = 5^_^6 for each group of mice).

(K) mRNA expression of *Qtrt2* in livers of LKO mice and control (f/f, which was also referred to WT), was measured using qRT-PCR and expressed relative to *Tbp* as fold change over control (n = 3 for each group of mice).

(L) mRNA expression of *Qtrt1* in different tissues of LKO mice and their littermate control, was measured using qRT-PCR and expressed relative to different internal reference gene (*Tbp* for liver, *Hprt* for colon, and *Gapdh* for other tissues) as fold change over control (n = 3 for each group of mice).

(M) Protein expression of Qtrt1 in liver of LKO mice and control ( n = 4 for each group of mice).

(N) Protein expression of Qtrt1 in other tissues of LKO mice and control ( n = 1 for each group of mice).

Data are presented as the mean ± SEM. Statistical significance (*p < 0.05, **p < 0.01; *n.s.* or ns, not significant) was determined using Mann-Whitney U test for K and unpaired t-tests for others.


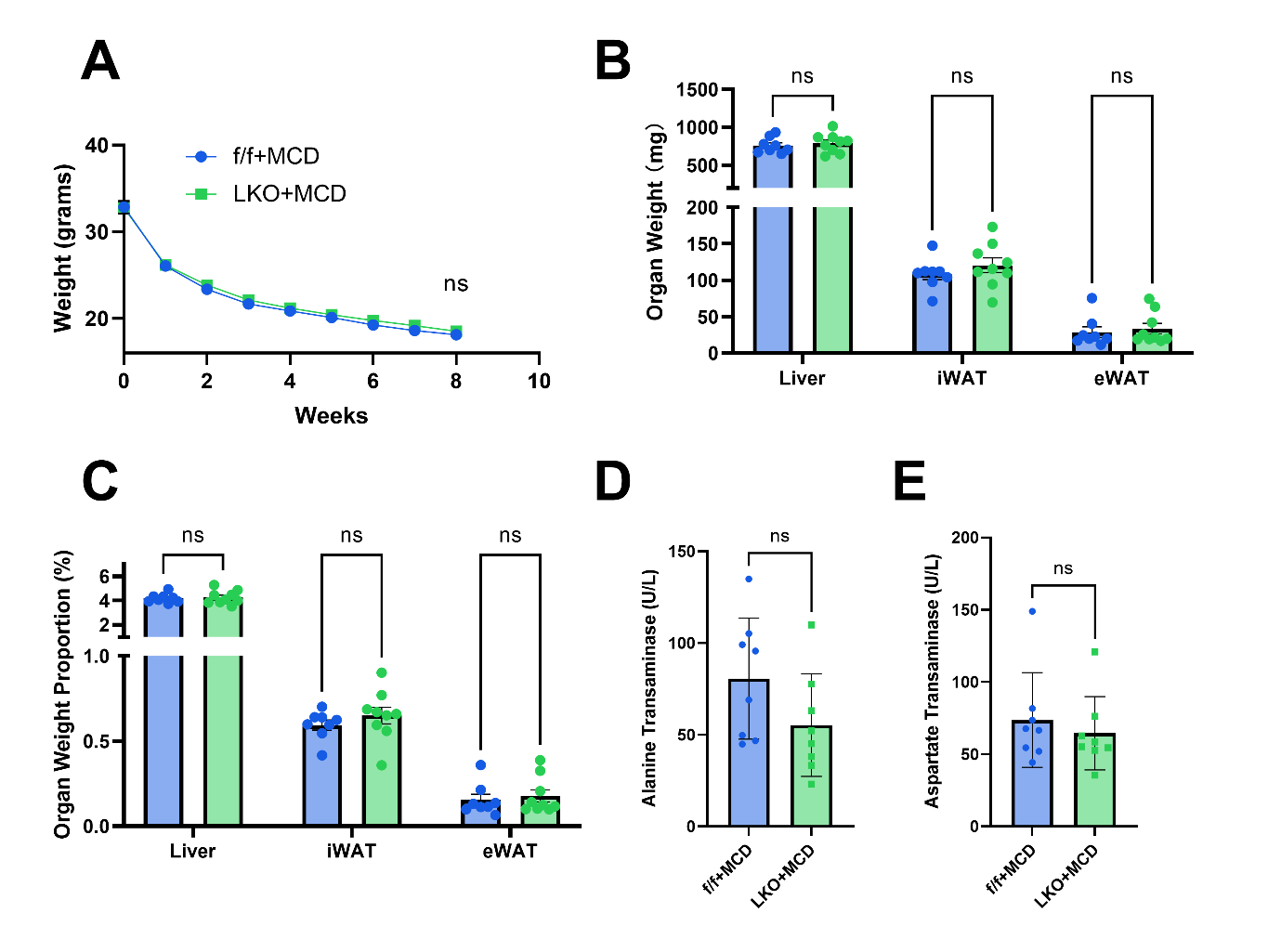


**Supplementary Fig. 2. Liver-specific QTRT1 deficiency did not impact bodyweight, organ mass or transaminase levels in circulation when fed a methionine- and choline-deficient diet (MCD).**

(A) Bodyweight of mouse cohorts fed with a MCD diet for 8 weeks (n = 8^_^9 for each group of mice).

(B-C) Organ mass at sacrifice and mass ratio calculated by the proportion of organ mass in mouse cohorts at euthanization (n = 8^_^9 for each group of mice).

(D-E) Serum ALT and AST levels in LKO mice and their counterpart control mice at euthanization (n = 8 for each group of mice).

Data are presented as the mean ± SEM. Statistical significance (ns, not significant) was determined using an unpaired t-test.


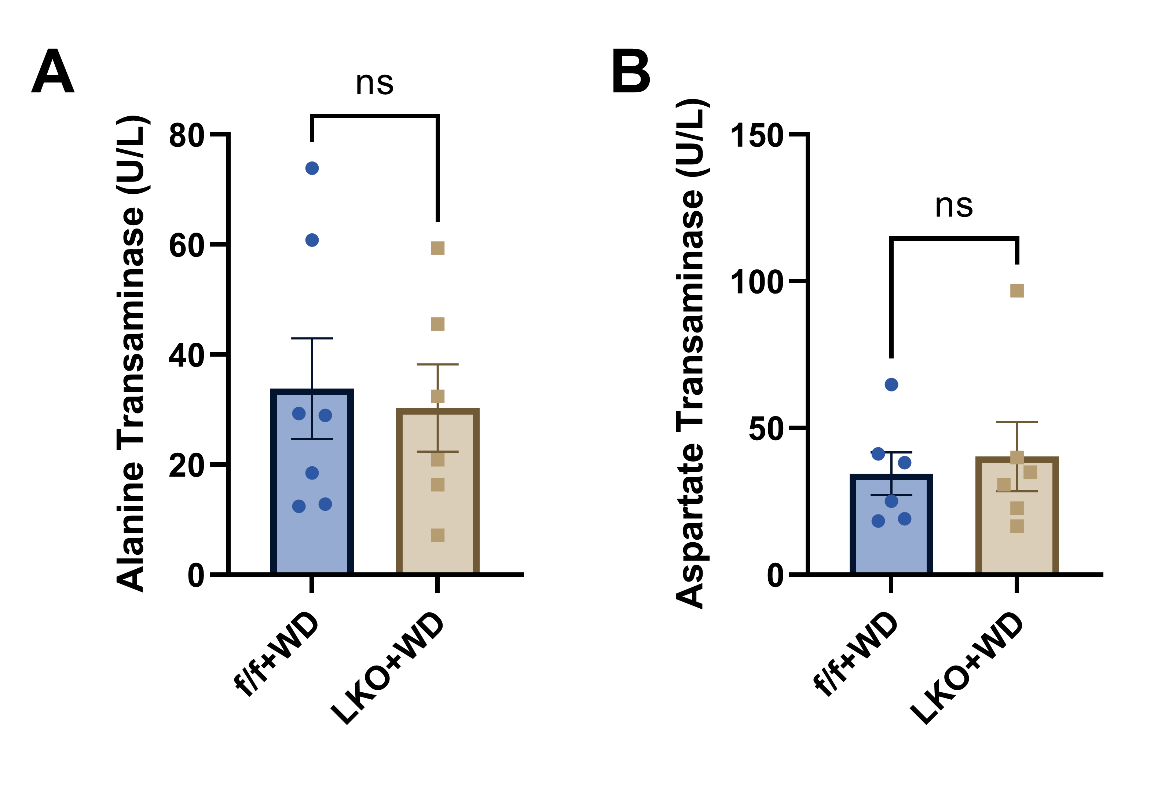


**Supplementary Fig. 3. Liver-specific QTRT1 deficiency did not impact transaminase levels in circulation when fed a Western Diet (WD).**

(A-B) Serum ALT and AST levels in LKO mice and their counterpart control mice at euthanization (n = 6^_^7 for each group of mice).

Data are presented as the mean ± SEM. Statistical significance (ns, not significant) was determined using an unpaired t-test.


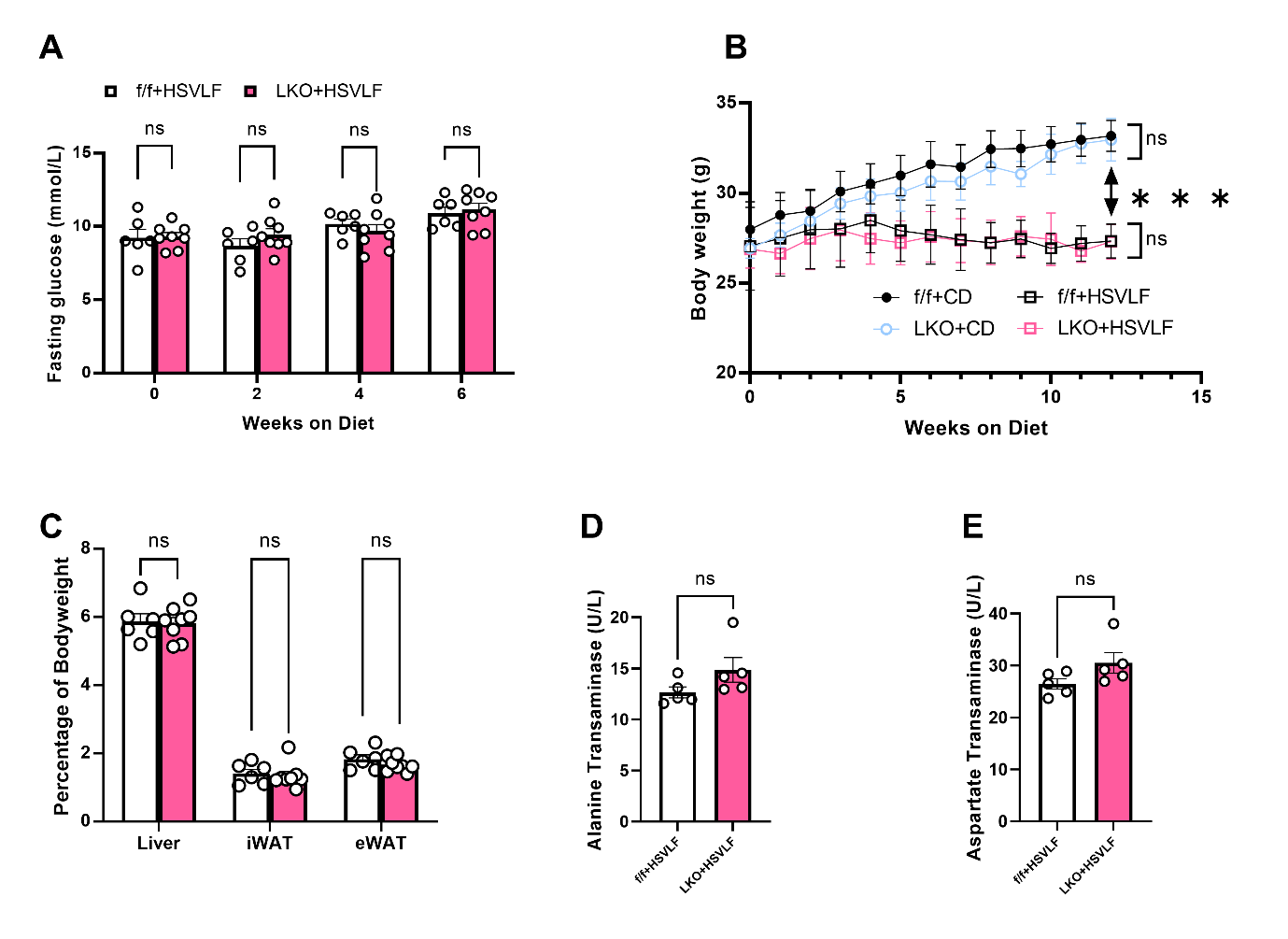


**Supplementary Fig. 4. Liver-specific QTRT1 deficiency did not change fasting glucose levels, bodyweight and organ mass, or transaminase levels in circulation when fed a high-sucrose and very low-fat diet (HSVLF).**

(A) Fasting glucose levels in f/f and LKO mice at different time points when fed a HSVLF diet (n = 6^_^8 for each group of mice).

(B) Bodyweight curve of f/f and LKO mice fed with HSVLF and chow diet (CD) for 12 weeks (n = 6^_^8 for each group of mice).

(C) Organ mass ratio of LKO mice at euthanization (n = 6^_^8 for each group of mice).

(D-E) Serum ALT and AST levels in LKO mice and their counterpart control mice at euthanization (n = 6^_^8 for each group of mice).

Data are presented as the mean ± SEM. Statistical significance (***p < 0.001; ns, not significant) was determined using an unpaired t-test.


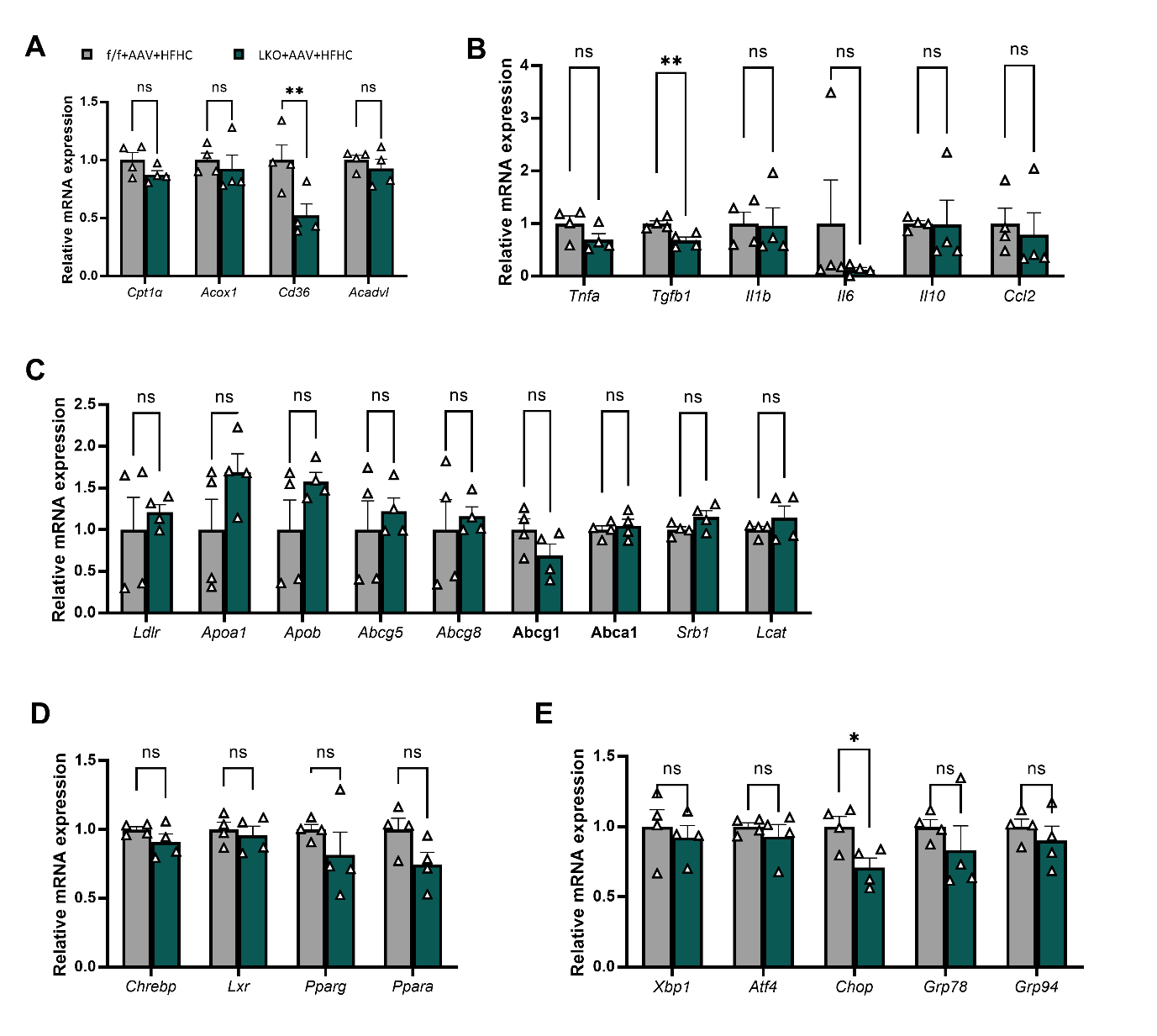


**Supplementary Fig. 5. QTRT1 deficiency resulted in mild changes in gene expression related to fatty acid oxidation, inflammatory cytokine production, lipoprotein transportation, other upstream transcription factors and ER stress factors.**

(A) mRNA expression of genes related to fatty acid uptake and oxidation in the livers of LKO mice injected with PCSK9-AAVs and fed an HFHC diet for 12 weeks, was measured using qRT-PCR and expressed relative to *Tbp* and as fold change over control as follows (n = 4 for each group of mice).

(B) mRNA expression levels of inflammatory cytokine genes in the livers at euthanization (n = 4 for each group of mice).

(C) mRNA expression of genes involved in lipid transportation (n = 4 for each group of mice).

(D) mRNA expression of genes of transcription factors other than SREBPs regulating lipid synthesis and transportation (n = 4 for each group of mice).

(E) mRNA expression of genes encoding critical factors involved in ER stress (n = 4 for each group of mice).

Data are presented as the mean ± SEM. Statistical significance (*p < 0.05, **p < 0.01; ns, not significant) was determined using unpaired t-tests.


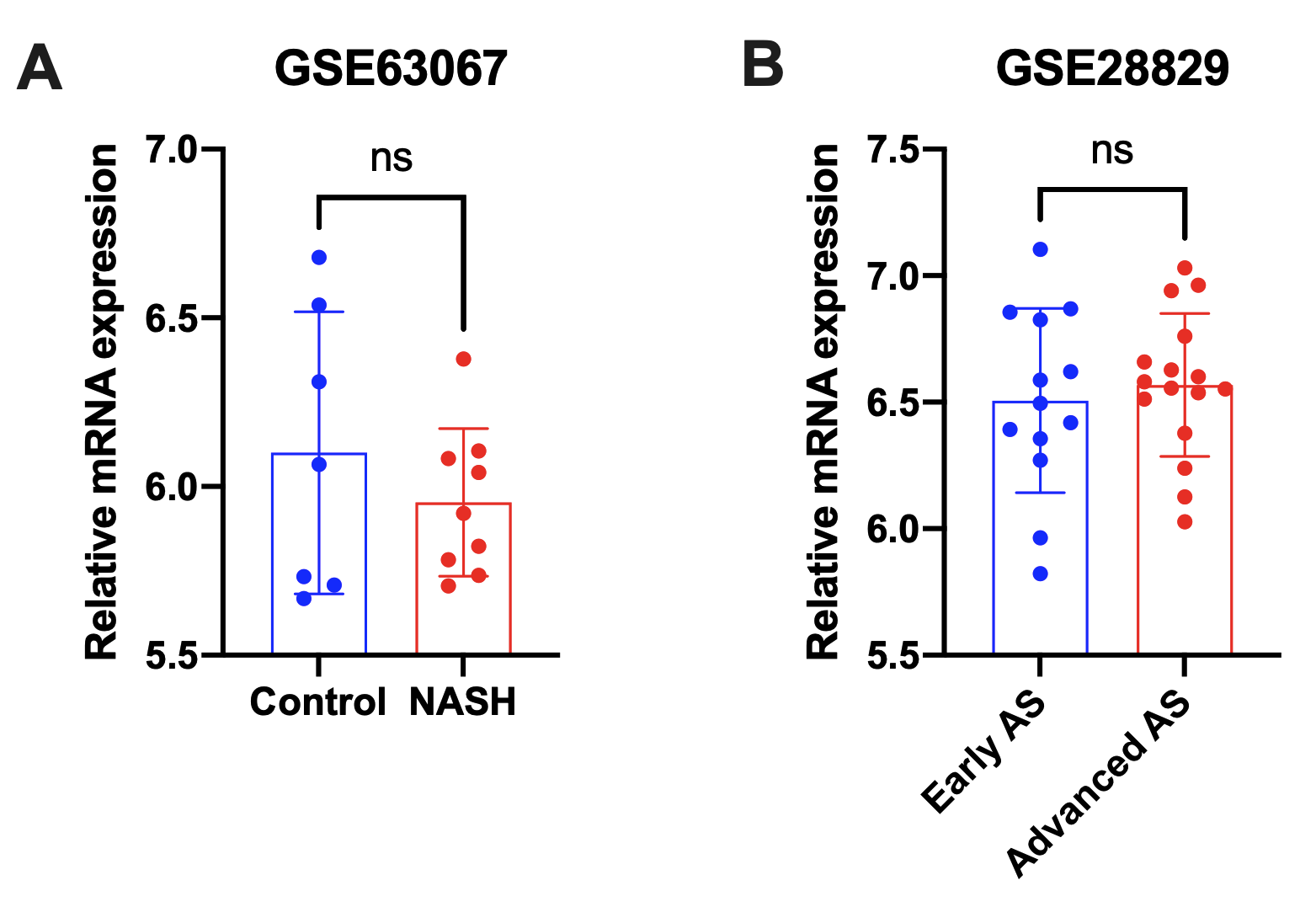


**Supplementary Fig. 6. Relative gene expression of *QTRT1* in human biosamples of fatty liver disease and atherosclerotic disease.**

(A) The relative expression of *QTRT1* in liver samples of healthy control participants versus non-alcoholic steatohepatitis (NASH) patients in public datasets (GSE63067 (control: n = 7; NASH: n = 9)).

(B) The relative expression of *QTRT1* in early atherosclerotic (AS) lesions in carotid artery segments versus advanced lesions in public datasets from patients (GSE28829 (early AS: n = 13; advanced AS: n = 16)).

Data are presented as the mean ± SD. Statistical significance (ns, not significant) was determined using unpaired t-tests.


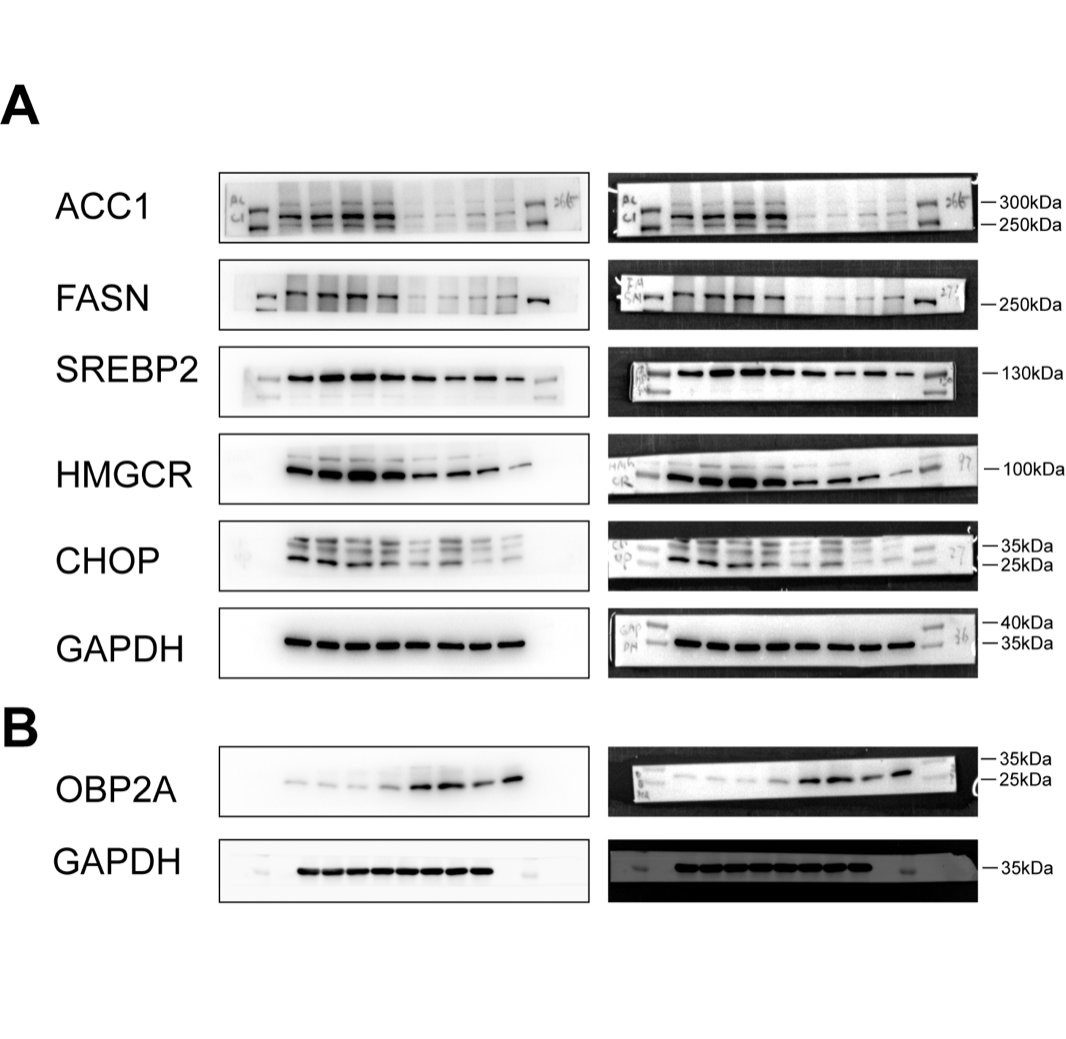


**Supplementary Fig. 7. Original full immunoblotting images of each tested protein in Fig. 1K and N, with related molecular weight markers.**

(A-B) Full images of immunoblotting (left: images of blots; right: merged images of blots and bright field with molecular weight markers labeled) of main genes regulating lipid synthesis and endoplasmic reticulum stress, and OBP2A in LKO mice injected with PCSK9 AAVs and fed with HFHC diet; n = 4 for each group of mice.

**Primers used for real-time PCR.**

| Primer name | Sequence（5′ to 3′） |
| --- | --- |
| mTbp-F | ACCCTTCACCAATGACTCCTATG |
| mTbp-R | TGACTGCAGCAAATCGCTTGG |
| mGapdh-F | AGGTCGGTGTGAACGGATTTG |
| mGapdh-R | TGTAGACCATGTAGTTGAGGTCA |
| mHprt-F | TCAGTCAACGGGGGACATAAA |
| mHprt-R | GGGGCTGTACTGCTTAACCAG |
| mQtrt1-F | CAGCACTACACCACCTAACCGT |
| mQtrt1-R | ACAGAGGCTGTGGTCACCATAC |
| mQtrt2-F | ATCAAAGTCGTTAATGGCTGTCG |
| mQtrt2-R | CCAGTCCTGGTGTAGAGAAGAC |
| mSrebp1c-F | TGACCCGGCTATTCCGTGA |
| mSrebp1c-R | CTGGGCTGAGCAATACAGTTC |
| mAcc1-F | GCCTCTTCCTGACAAACGAG |
| mAcc1-R | TGACTGCCGAAACATCTCTG |
| mScd1-F | CCTCCTGCAAGCTCTACACC |
| mScd1-R | CAGCCGAGCCTTGTAAGTTC |
| mFasn-F | CTGACTCGGCTACTGACACG |
| mFasn-R | TGAGCTGGGTTAGGGTAGGA |
| mDgat1-F | GATTGTGGGCCGATTCTTCC |
| mDgat1-R | CATACATGAGCACAGCCACC |
| mLdlr-F | TCAGACGAACAAGGCTGTCC |
| mLdlr-R | CCATCTAGGCAATCTCGGTCTC |
| mApoa1-F | GCTCAAGAGCAACCCTACCTT |
| mApoa1-R | GCTTTCTCGCCAAGTGTCTTC |
| mApob-F | TTGGCAAACTGCATAGCATCC |
| mApob-R | TCAAATTGGGACTCTCCTTTAGC |
| mAbcg1-F | CTTTCCTACTCTGTACCCGAGG |
| mAbcg1-R | CGGGGCATTCCATTGATAAGG |
| mAbca1-F | GCTTGTTGGCCTCAGTTAAGG |
| mAbca1-R | GTAGCTCAGGCGTACAGAGAT |
| mLxr-F | CTCAATGCCTGATGTTTCTCCT |
| mLxr-R | TCCAACCCTATCCCTAAAGCAA |
| mAbcg5-F | AGGGCCTCACATCAACAGAG |
| mAbcg5-R | GCTGACGCTGTAGGACACAT |
| mAbcg8-F | CTGTGGAATGGGACTGTACTTC |
| mAbcg8-R | GTTGGACTGACCACTGTAGGT |
| mSrb1-F | TTTGGAGTGGTAGTAAAAAGGGC |
| mSrb1-R | TGACATCAGGGACTCAGAGTAG |
| mLcat-F | GTAACCACACACGGCCTGTC |
| mLcat-R | TCTTACGGTAGCACATCCAGTT |
| mSrebp2-F | GCAGCAACGGGACCATTCT |
| mSrebp2-R | CCCCATGACTAAGTCCTTCAACT |
| mHmgcr-F | AGCTTGCCCGAATTGTATGTG |
| mHmgcr-R | TCTGTTGTGAACCATGTGACTTC |
| mAcadvl-F | CTACTGTGCTTCAGGGACAAC |
| mAcadvl-R | CAAAGGACTTCGATTCTGCCC |
| mAcox1-F | CAGGAAGAGCAAGGAAGTGG |
| mAcox1-R | CCTTTCTGGCTGATCCCATA |
| mCpt1α-F | TGCACTACGGAGTCCTGCAA |
| mCpt1α-R | GGACAACCTCCATGGCTCAG |
| mCd36-F | AAGCTATTGCGACATGATT |
| mCd36-R | GATCCGAACACAGCGTAGAT |
| mChrebp-F | AGATGGAGAACCGACGTATCA |
| mChrebp-R | ACTGAGCGTGCTGACAAGTC |
| mPparg-F | CTTGGCTGCGCTTACGAAGA |
| mPparg-R | GAAAGCTCGTCCACGTCAGAC |
| mPpara-F | GAACGGCTTCCTCAGGTTCTT |
| mPpara-R | GCGTACGGCAATGGCTTTAT |
| mTnfa-F | CAGCGCTGAGGTCAATCTGCC |
| mTnfa-R | TGCCCGGACTCCGCAA |
| mTgfb1-F | TTGCTTCAGCTCCACAGAGA |
| mTgfb1-F | TGGTTGTAGAGGGCAAGGAC |
| mIl1b-F | GAAATGCCACCTTTTGACAGTG |
| mIl1b-R | TGGATGCTCTCATCAGGACAG |
| mIl6-F | ATCCAGTTGCCTTCTTGGGACTGA |
| mIl6-R | TAAGCCTCCGACTTGTGAAGTGGT |
| mIl10-F | GCTCTTACTGACTGGCATGAG |
| mIl10-R | CGCAGCTCTAGGAGCATGTG |
| mIl-18-F | ACAGGCCTGACATCTTCTGC |
| mIl-18-R | CCTTGAAGTTGACGCAAGAGT |
| mCcl2-F | TCACCTGCTGCTACTCATTCACCA |
| mCcl2-R | TACAGCTTCTTTGGGACACCTGCT |
| mXbp1-F | GGTCTGCTGAGTCCGCAGCAGG |
| mXbp1-R | AGGCTTGGTGTATACATGG |
| mAtf4-F | AAGGAGGAAGACACTCCCTCT |
| mAtf4-R | CAGGTGGGTCATAAGGTTTGG |
| mChop-F | CATACACCACCACACCTGAAAG |
| mChop-R | CCGTTTCCTAGTTCTTCCTTGC |
| mGrp78-F | TGGTATTCTCCGAGTGACAGC |
| mGrp78-R | AGTCTTCAATGTCCGCATCC |
| mGrp94-F | GTTCGTCAGAGCTGATGATGAA |
| mGrp94-R | GCGTTTAACCCATCCAACTGAAT |
| mHspa1a-F | TGGTGCAGTCCGACATGAAG |
| mHspa1a-R | GCTGAGAGTCGTTGAAGTAGGC |
| mObp2a-F | GTCATTCGGGATGGGAAAG |
| mObp2a-R | GCTGTTGCAGACCTGGGTA |
| hTBP-F | CCACTCACAGACTCTCACAAC |
| hTBP-R | CTGCGGTACAATCCCAGAACT |
| hGAPDH-F | GTCTCCTCTGACTTCAACAGCG |
| hGAPDH-R | ACCACCCTGTTGCTGTAGCCAA |
| hQTRT1-F | GTAGTCTGCGTGGCTCTTGGAT |
| hQTRT1-R | GCCGAAGTCCTTCTCAAACACC |
| hSREBP1c-F | CACTGGTCGTAGATGCGGAGAA |
| hSREBP1c-R | TCATTGATGGAGGAGCGGTAGC |
| hACC1-F | CTGGCTGGCTGGACAGACTGAT |
| hACC1-R | ACGCTATTCCGCAGGCTCACA |
| hSCD1-F | TTCCTACCTGCAAGTTCTACACC |
| hSCD1-R | CCGAGCTTTGTAAGAGCGGT |
| hFASN-F | TTCTACGGCTCCACGCTCTTCC |
| hFASN-R | GAAGAGTCTTCGTCAGCCAGGA |
| hHSPA1A-F | TGTAACCCCATCATCAGCGG |
| hHSPA1A-R | GAAGCTCCAAAACAAAAACAGCA |
| hOBP2A-F | TAAGGACTTTCCGGAGGACA |
| hOBP2A-R | TCCGCATCAGGATTTTCTTC |
| hCHREBP-F | AGTGCTTGAGCCTGGCCTAC |
| hCHREBP-R | TTGTTCAGGCGGATCTTGTC |
| hPPARG-F | TACTGTCGGTTTCAGAAATGCC |
| hPPARG-R | GTCAGCGGACTCTGGATTCAG |
| hLXR-F | TCTGGAGACATCTCGGAGGTA |
| hLXR-R | GGCCCTGGAGAACTCGAAG |

**References：**

1. Chen MJ, Xu YT, Sun L, et al. A novel mouse model of familial combined hyperlipidemia and atherosclerosis. *Acta Pharmacol Sin.* 2024;45(6):1316-1320.

2. Goettsch C, Hutcheson JD, Hagita S, et al. A single injection of gain-of-function mutant PCSK9 adeno-associated virus vector induces cardiovascular calcification in mice with no genetic modification. *Atherosclerosis.* 2016;251:109-118.

3. Tamargo IA, Baek KI, Xu C, et al. HEG1 Protects Against Atherosclerosis by Regulating Stable Flow-Induced KLF2/4 Expression in Endothelial Cells. *Circulation.* 2024;149(15):1183-1201.

4. Mridha AR, Wree A, Robertson AAB, et al. NLRP3 inflammasome blockade reduces liver inflammation and fibrosis in experimental NASH in mice. *J Hepatol.* 2017;66(5):1037-1046.

5. Latif MU, Schmidt GE, Mercan S, et al. NFATc1 signaling drives chronic ER stress responses to promote NAFLD progression. *Gut.* 2022;71(12):2561-2573.

6. Miyazaki M, Flowers MT, Sampath H, et al. Hepatic stearoyl-CoA desaturase-1 deficiency protects mice from carbohydrate-induced adiposity and hepatic steatosis. *Cell Metab.* 2007;6(6):484-496.

7. Qu W, Zhou X, Jiang X, et al. Long Noncoding RNA Gpr137b-ps Promotes Advanced Atherosclerosis via the Regulation of Autophagy in Macrophages. *Arterioscler Thromb Vasc Biol.* 2023;43(11):e468-e489.

8. Morrow MR, Batchuluun B, Wu J, et al. Inhibition of ATP-citrate lyase improves NASH, liver fibrosis, and dyslipidemia. *Cell Metab.* 2022;34(6):919-936.e918.

9. Yao K, Dai Y, Shen J, et al. Exome sequencing identifies rare mutations of LDLR and QTRT1 conferring risk for early-onset coronary artery disease in Chinese. *Natl Sci Rev.* 2022;9(8):nwac102.

10. Ding Z, Wei Y, Dai J, et al. Deficiency of SDHC promotes metastasis by reprogramming fatty acid metabolism in colorectal cancer. *J Transl Med.* 2024;22(1):544.
